# Platelets promote breast cancer cell MCF-7 metastasis by direct interaction: surface integrin α2β1-contacting-mediated activation of Wnt-β-catenin pathway

**DOI:** 10.1186/s12964-019-0464-x

**Published:** 2019-11-07

**Authors:** Xiao-xiao Zuo, Ya Yang, Yue Zhang, Zhi-gang Zhang, Xiao-fei Wang, Yong-gang Shi

**Affiliations:** Department of Radiation Oncology, The First Affiliated Hospital of Zhengzhou University, No.1 East Jianshe Road, Erqi District, Zhengzhou, Henan Province 450000 People’s Republic of China

**Keywords:** Epithelial-mesenchymal transition, Platelet, Direct interaction, MCF-7 cell, Wnt-β-catenin, TGF-β1

## Abstract

**Background:**

Integrin-mediated platelet-tumor cell contacting plays an important role in promoting epithelial-mesenchymal transition (EMT) transformation of tumor cells and cancer metastasis, but whether it occurs in breast cancer cells is not completely clear.

**Objective:**

The purpose of this study was to investigate the role of integrin α2β1 in platelet contacting to human breast cancer cell line MCF-7 and its effect on the EMT and the invasion of MCF-7 cells.

**Methods:**

Human platelets were activated by thrombin, and separated into pellets and releasates before the co-incubation with MCF-7 cells. Cell invasion was evaluated by transwell assay. The surface integrins on pellets and MCF-7 cells were inhibited by antibodies. The effect of integrin α2β1 on Wnt-β-catenin pathway was assessed by integrin α2β1-silencing and Wnt-β-catenin inhibitor XAV. The therapeutic effect of integrin α2β1-silencing was confirmed in the xenograft mouse model.

**Results:**

Pellets promote the invasion and EMT of MCF-7 cells via direct contacting of surface integrin α2β1. The integrin α2β1 contacting activates Wnt-β-catenin pathway and promotes the expression of EMT proteins in MCF-7 cells. The activated Wnt-β-catenin pathway also promotes the autocrine of TGF-β1 in MCF-7 cells. Both Wnt-β-catenin and TGF-β1/pSmad3 pathways promote the expression of EMT proteins. Integrin α2β1-silencing inhibits breast cancer metastasis in vivo*.*

**Conclusions:**

The direct interaction between platelets and tumor cells exerts its pro-metastatic function via surface integrin α2β1 contacting and Wnt-β-catenin activation. Integrin α2β1-silencing has the potential effect of inhibiting breast cancer metastasis.

## Background

Platelets, as small cell fragments, are not only important coagulation-related factors, but also play a vital role in cancer metastasis [1]. The increased level of platelets was correlated with tumor progression, recurrence and distant metastasis in various cancer types [2, 3]. The high level of platelets also has a close correlation with poor recurrence-free survival rates and cancer-specific survival rates [4, 5]. The interaction between platelets and tumor cells activates signaling pathways, such as the Wnt-β-catenin pathway, and promotes the expression of epithelial-mesenchymal transition (EMT) proteins, thus making tumor cells change to a more aggressive phenotype [6]. In breast cancer, Ishikawa et al. found that the accumulation of platelets in primary tumor cells was positively correlated with chemoresistance, and can induce EMT of breast cancer cells [7]. Therefore, inducing EMT transformation of tumor cells is the major way of platelets promoting cancer metastasis.

Previous study mainly investigated the indirect interactions between platelets and tumor cells——secreting releasates (mainly α-granules) of platelets to promote EMT of tumor cells [8]. It has been reported that platelet-derived α-granules containing transforming growth factor-β1 (TGF-β1) increased the growth of primary tumors in murine models of ovarian cancer [9, 10]. However, in recent years, the effect of direct interaction of platelet and tumor cells on promoting EMT has been attracting attentions in the field of cancer treatment. Pang et al. demonstrated that the direct interaction between tumor cells and platelet fractions, named as pellets (with the activation marker of P-selectin, but without releasates [11]), promotes the EMT and extracellular matrix (ECM) degradation, inducing a more aggressive phenotype in tumor cells [12]. Labelle et al. demonstrated that direct platelet-tumor cell contacting and platelet-derived TGF-β1 synergistically activate the TGF-β1/Smad pathway in cancer cells, resulting in their EMT transformation and enhanced metastasis [13]. Whereas, the mechanism underlying the direct interaction between platelets and breast cancer cells is largely unknown.

Integrins mediate cell-to-cell contacting, and are key mediators in delivering signals between cells. They were discovered by Hynes RO and his colleagues [14, 15]. Also, integrins are suggested to be involved in the tumor growth and progression [16], and were regarded as a therapeutic target for tumors. As reported, the combined blockade of both tumor integrin αvβ3 and platelet integrin αIIbβ3 inhibited platelet-cell contacting and suppressed angiogenesis and tumor growth of melanoma [17]. α2β1, a kind of integrin, has been shown to exist in the surface of breast cancer cells, and is believed to mediate the direct interaction between tumor cells and platelets [18]. However, how integrin α2β1 mediates the EMT by platelet-breast cancer cell contacting remains unknown.

Herein, this current study defined the role of integrin α2β1 in mediating the direct interaction between platelets and breast cancer cells, and explored its effect on the promotion of EMT by controlling the correlated signaling molecules, thus implying the important role of direct contacting of platelets and breast cancer cells in promoting breast cancer metastasis.

## Materials and methods

### Preparation of platelets and platelet fractions

Platelets and platelet fractions used in this study were collected from human blood as previously described [13]. Briefly, healthy human blood was centrifuged at 250G for 10 min. The plasma and buffy coat were gently transferred to a fresh tube, and the centrifugation was repeated at 2000G for 10 min. The platelet-rich plasma in the bottom was then washed with the platelet washing solution for 3 times. To prepare platelet fractions, platelets were stimulated by thrombin 0.5 U/ml for 15 min at 37 °C. The pellets (platelet membranes) were separated from the releasates (supernatant containing active components) by centrifugation at 2800G for 7 min. To determine the extent of contamination of the thrombin-activate pellets with soluble platelet proteins, platelet factor 4 (PF4) was measured using an ELISA assay (Abcam, Cambridge, UK) and compared with the PF4 levels in the releasates and whole platelets solubilised by the addition of 0.1% Triton-X 100 [12].

### P-selectin exposure analysis

A minimum of 10,000 gated events was analyzed on a FACScalibur flow cytometer (BD Biosciences, San Jose, CA, US). The anti-CD41 conjugated with fluorescein (FITC, BD Pharmingen, San Jose, CA) and the anti-CD62P conjugated with phycoerythrin (PE, BD Pharmingen) were used as antibodies. Then they were added to the activated platelets with a final dilution of 1:20 (v/v). FITC and PE-conjugated isotype-matched antibodies were used to control nonspecific labeling of antibodies. The samples were fixed with 0.4% paraformaldehyde and analyzed by flow cytometry after antibody incubation for 15 min at 37 °C.

### Transwell invasion assay

Human breast cancer cell lines, MCF-7, MDA-MB-231, and SK-BR-3 (all from American Type Culture Collection, ATCC, Manassas, VA, US), were maintained at 37 °C, 5% CO2, grown in Dulbecco’s Modified Eagle’s medium (DMEM; ThermoFisher, Pittsburgh, PA, US) containing 1% penicillin-streptomycin (Gibco, NY, USA) and 10% fetal bovine serum (FBS; ThermoFisher) at pH 7.2 overnight. The invasion assay was performed in 24-well BD Biocoat™ Matrigel™ Invasion Chambers (8 μm pore size; BD Biosciences) [13]. Total 5 × 10^4^ MCF-7 cells were plated in transwell inserts. Then the DMEM buffer (*the buffer group*), thrombin-activated platelets (*the platelet group*), releasates (*the releasate group*), pellets (*the pellet group*), and the supernatant of the co-incubation of pellets with MCF-7 cells for 48 h [*the pellet + MCF-7 conditioned medium (CM) group*] were added. Both upper and lower chambers contained DMEM. After 48 h, cells remaining in the upper part of the transwell were removed with a cotton swab. Migrated cells were then stained with Crystal Violet 0.5% and the total number of cells was counted with a Zeiss Axiovert 200 microscope (Zaventem, Belgium). The invasion assay was also performed in MDA-MB-231 cells and SK-BR-3 cells after adding platelets.

### Direct interaction between tumor cells and platelets or platelet fractions

The medium of MCF-7 cells was changed for fresh DMEM immediately prior to the treatment. Then the DMEM buffer (*the buffer group*), thrombin-activated platelets (*the platelet group*), releasates (*the releasate group*), pellets (*the pellet group*), and the supernatant of the co-incubation of pellets with MCF-7 cells for 48 h (*the pellet + MCF-7 CM group*) were added, and incubated for 40 h.

### Quantitative real-time PCR (qRT-PCR)

Total RNAs were extracted from the tumor cell line or tumor tissues using isolation kit according to the manufacturer’s protocol. The cDNA was generated from RNAs using a cDNA Reverse Transcription Kit (Applied Biosystems, ThermoFisher). The expression of RNAs was analyzed by qRT-PCR using Power SYBR Green PCR Master Mix (Applied Biosystems, ThermoFisher) with GAPDH as endogenous controls. Relative expression levels of all genes were calculated as 2^–ΔΔCt^. The primers used were shown in Table 1.

**Table 1:** Primers and promoter sequences used in qRT-PCR and ChIP

qRT-PCR Primers (from 5′ to 3′)	
Snail	F: CTTCCAGCAGCCCTACGAC
	R: CGGTGGGGTTGAGGATCT
Slug	F: AGATGCATATTCGGACCCAC
	R: CCTCATGTTTGTGCAGGAGA
E-cadherin	F: CGACCCAACCCAAGAATCTA
	R: AGGCTGTGCCTTCCTACAGA
Fibronectin	F: CCATCGCAAACCGCTGCCAT
	R: AACACTTCTCAGCTATGGGCTT
COL1A1	F: TAACTTCTGGACTATTTGCGGACTTTTTGG
	R: GGGCGAGGGAGGAGAGAA
MMP9	F: CCTGGAGACCTGAGAACCAATC
	R: GATTTCGACTCTCCACGCATC
tgfb1	F: CGTGGAGCTGTACCAGAAATA
	R: TCCGGTGACATCAAAAGATAA
GAPDH	F: TGTTCGACAGTCAGCCGC
	R: GGTGTCTGAGCGATGTGGC
ChIP Primers (from 5′ to 3′)	
Snail promoter	F: CACTTCCTCTGGGAAGTCACC
	R: CCTTCCCTTATCCAGTGTTTACGGAG
Slug promoter	F: CTGCACCACATCTGGAAGCCAG
	R: CCAATCACAGCTGAGAGGTTCAG
tgfb1 promoter	F: GCAACTTCGACCGCTACGG
	R: CTGCGACCCCATACATTTACTG
GAPDH promoter	F: AGCTCAGGCCTCAAGACCTT
	R: AAGAAGATGCGGCTGACTGT

### Western blotting

Total protein was extracted from the tumor cell line or tumor tissues using RIPA lysis buffer. The concentration of total protein was quantified using a BCA kit (ThermoFisher). The protein samples (20 μg/sample) were separated by SDS-PAGE and then transferred to poly-vinylidene difluoride (PVDF) membranes (Merck Millipore, Danvers, MA, US). After blocking with 5% fat-free milk in Tris-buffered saline containing 0.1% Tween 20 (TBST) for 1 h, the proteins were immunoblotted with primary antibodies at 4 °C overnight. After washing with TBST three times, the proteins were incubated with horseradish peroxidase-conjugated secondary antibodies for 1 h at room temperature. After washing with TBST three times, protein signals were determined using a substrate chemiluminescence detection system (ThermoFisher) with the Image Lab Software (Bio-Rad, Hercules, CA, US). The primary antibodies used were anti-β-catenin (1:5000, Rabbit monoclonal antibody, Abcam, ab32572), anti-integrin α2 (1:10000, Rabbit monoclonal antibody, Abcam, ab133557), anti-β-actin (1:500, Mouse monoclonal antibody, Abcam, ab8226), anti-Lamin B1 (0.1 μg/ml, Rabbit polyclonal antibody, Abcam, ab16048), and anti-pSmad3 (1:2000, Rabbit monoclonal antibody, Abcam, ab52903).

### Analysis of tumor cell contacting to platelets or pellets

The platelet adhesion assay was performed to evaluate the contacting between platelets/pellets and tumor cells as previously described [18]. Briefly, 5 × 10^4^ cells/ml breast cancer cells were seeded in a 96-well plate. Washed thrombin-activated platelets or pellets labeled with fluorescein (FITC, BD Pharmingen) were added to the cells for 30 min at 37 °C. The non-adherent platelets or pellets were discarded. Then the flow cytometry was used to perform the platelet/pellet adhesion assay.

### Antibody inhibition of pellet or tumor cell membrane

The antibody inhibition of pellet and tumor cell membrane was performed as previously described [12]. In brief, the 5 × 10^8^ CFDA-SE-conjugated thrombin-activated pellet were pre-incubated with 50μg/ml anti-P-selectin, anti-AK7 (integrin α2β1), or anti-CRC64 (integrin αIIbβ3) on ice for 30 min. Then they were added to 3 × 10^5^ MCF-7 cells in 100 μl of DMEM in a 96-well plate and incubated at 37 °C for 30 min. To prevent further binding before flow cytometry, 100 μl /well ice cold 0.1% BSA/PBS was added and the flow cytometry was performed immediately. The antibody inhibition of MCF-7 cell membrane was also performed as described above, and the antibodies used were anti-AK7 and anti-CRC64. The anti-AK7 was used to inhibit the cell surface integrin α2β1 of MDA-MB-231 cells and SK-BR-3 cells.

### Integrin α2β1-silencing MCF-7 cell line

The shRNA specific for the integrin α2β1 subunit (Sigma, St. Louis, MO, US) was cloned to the lentiviral plasmid vector pLKO.1-puro, and the pLKO.1-puro lentiviral vector without shRNA (empty vector) was used as the control [19]. Lentiviruses were produced in HEK293T cells by co-transfection of plasmid vector containing shRNA or control vector. Then the MCF-7 cells were infected with shRNA lentivirus or empty lentivirus in the presence of polybrene (8 mg/ml) and selected with puromycin (1–2 mg/ml) for 4–6 days.

### TGF-β1 ELISA

TGF-β1 levels were detected in MCF-7 cells co-cultured with DMEM (the buffer group), platelets, releasates, or pellets for 40 h with the Quantikine TGF-β1 immunoassay kit (R&D Systems, Minneapolis, MN, US).

### Chromatin Immunoprecipitation assay (ChIP)

The promoter of Snail and Slug binding by pSmad3 and/or β-catenin was determined by ChIP [20]. Briefly, after the co-culture with activated platelets with or without XAV (Wnt-β-catenin inhibitor) or SB (TGF-β1/Smad3 inhibitor), the MCF-7 cells were fixed with 1% formaldehyde, sonicated on ice to shear the DNA into the fragments from 200 bp to 500 bp. The lysate was subjected to immunoprecipitations with anti-pSmad3, anti-β-catenin, or non-specific rabbit IgG. The immunoprecipitated DNA was subjected to PCR to amplify a fragment of Snail, Slug, or tgfb1 promoter. The PCR products were run electrophoretically on a 1% agarose gel and visualized by ethidium bromide staining. The ChIP primers used were shown in Table 1.

### Immunoprecipitation (IP)

For pSmad3/β-catenin IP, three subconfluent 35-mm dishes of platelet-co-cultured MCF-7 cells were each extracted with 1 ml each of PBS containing 0.1% Triton X-100, 1:1000 CLAP, 2.5 mM sodium pyrophosphate, 1 mM β-glycerophosphate, and 1 mM sodium vanadate. The dishes were rocked at 4 °C for 15 min, and the cell lysates were centrifuged for 10 min at 4 °C. Then the precipitate was reextracted in a volume of 3 ml radioimmunoprecipitation assay buffer for 15 min at 4 °C. The supernatants were pooled and divided into 500-μl aliquots after centrifugation for 10 min. Each aliquot was then incubated with appropriate combinations of immune and preimmune sera and protein A–Sepharose beads for 14 h at 4 °C. The beads were collected by brief centrifugation and washed three times in PBS containing 0.1% Triton X-100 and once in PBS alone. Then the beads were suspended in SDS-PAGE sample buffer, heated to 95 °C for 5 min, and analyzed by western blotting.

### Promoter activity

The promoter activity was determined by the luciferase reporter assay. In brief, the promoter sequences of *tgfb1*, *Snail*, and *Slug* were cloned into the pGL3 vector. The MCF-7 cells with different treatments were transiently transfected with the vectors carrying *tgfb1*, *Snail*, or *Slug* promoter sequences using Lipofectamine 2000 (Invitrogen, Pittsburgh, PA, US). After 48 h of transfection, luciferase activity was detected using a luciferase reporter assay system (Promega, Madison, WI) according to the manufacturer’s protocol and was normalized to the renilla luciferase activity.

### Pulmonary metastasis assays

MCF-7 cells were co-incubated with platelets (*the MCF-7 + platelet group*) or without platelets (*the MCF-7 group*) for 40 h. MCF-7 cells which were stably interfering integrin α2β1 (Si-MCF-7) were obtained using the lentivirus vectors. The Si-MCF-7 were co-incubated with platelets (*the Si-MCF-7 + platelet group*) or without platelets (*the Si-MCF-7 group*) for 40 h. On the other hand, following the treatment of anti-AK7 for 30 min, the platelets were then co-incubated with MCF-7 cells for 40 h (*the MCF-7 + platelet/AK7 group*). The co-incubation between anti-AK7-untreated platelets and MCF-7 cells was the control (*the MCF-7 + platelet group*). The tumor cell monolayer was washed three times to remove platelets and dead cells. Then the tumor cells (1.5 × 10^6^ cells/100 μl PBS) were intravenously injected into the lateral tail vein of immunodeficient nude mice (6-week old, female). There were 5 mice in each group.

MDA-MB-231 cells were co-incubated with platelets for 40 h (*the MDA-MB-231 + Platelet group*). Following the simultaneous inhibition of surface integrin α2β1, MDA-MB-231/AK7 cells were co-incubated with platelet/AK7 for 40 h (*the MDA-MB-231/AK7 + platelet/AK7 group*). MDA-MB-231 cells cultured in the normal medium was the control (*the MDA-MB-231 group*). The tumor cell monolayer was washed three times to remove platelets and dead cells. Then the tumor cells (1 × 10^6^ cells/100 μl PBS) were intravenously injected into the lateral tail vein of immunodeficient nude mice (6-week old, female). There were 5 mice in each group. The Balb/c nude mice used in this study were bought from Shanghai Lab Animal Research Center (Shanghai, China).

After 5 weeks, mice were sacrificed and the lungs were separated. For hematoxylin-eosin (HE) staining, the pulmonary nodules were fixed in 10% formaldehyde of NaCl/Pi buffer with a pH of 7.4. After being dehydrated in alcohol, they were embedded in paraffin. Paraffin blocks were sliced in 4 μm pieces and stained with HE.

### Statistical analysis

Statistical analysis was performed using SPSS version 18.0 (SPSS Inc., Chicago, IL, US) with a Student’s t-test or analysis of variance. The data were expressed as the mean ± standard deviation (SD). *P* < 0.05 was considered statistically significant. All experiments were performed in triplicate.

## Results

### Activated platelets promote MCF-7 EMT via direct contacting

**Fig. 1:**
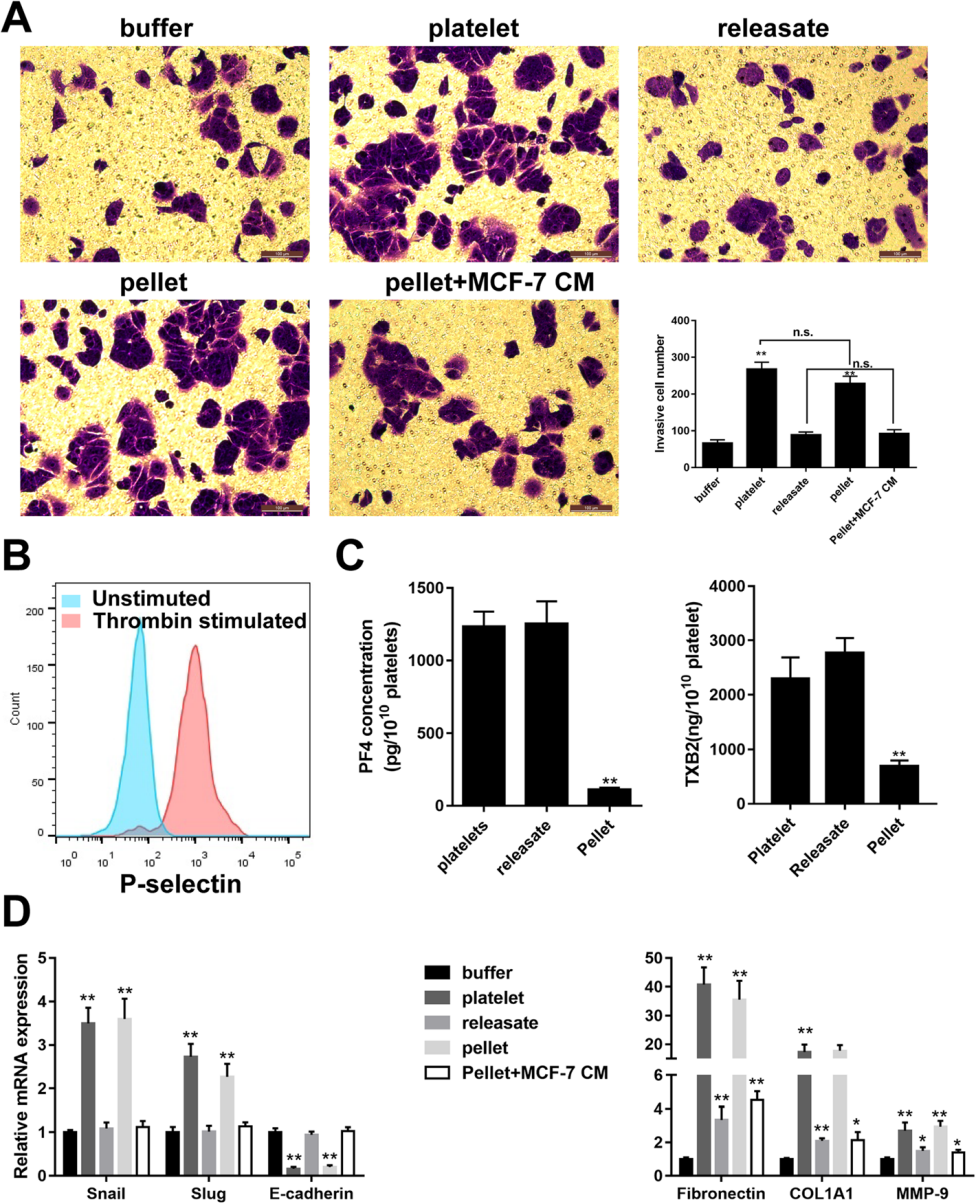
Direct contact between platelet and MCF-7 triggers the EMT of cells. Platelets were activated by thrombin and the platelet fractions, including pellets and releasates, were respectively separated. **a** The number of invasive MCF-7 cells was increased by platelet and pellet without being affected by releasates or conditioned medium (CM) obtained from the co-incubation of pellets and MCF-7 cells. Invasion experiment using the DMEM buffer was used as control. **b** The level of P-selectin exposure of the platelets before and after thrombin stimulation was analyzed by flow cytometry. **c** The concentration of PF4 (the marker of α-granules) and TXB2 (the products of functional platelets) were detected by ELISA assay. **d** The relative mRNA levels of EMT markers and ECM-associated molecules were detected by qRT-PCR. Scale bar = 100 μm.*p < 0.05, **p < 0.01

Platelet and platelet fractions, including releasates (active components of activated platelets) and pellets (platelet membranes without active components), were prepared from human blood and used to perform the transwell invasion assay, aiming at evaluating their impact on the invasion ability of breast cancer cells. The results showed that both platelets and pellets promoted the invasion of MCF-7 cells, but releasates did not significantly affect the invasion of MCF-7 cells (Fig. 1a). The P-selectin, an indicator for the assessment of platelet activation [21, 22] was analyzed by flow cytometry, and confirmed the activation of platelets and pellets (Fig. 1b). The concentration of PF4 (the marker of α-granules) and TXB2 (the products of functional platelets), were also detected. The result revealed that the PF4 and TXB2 content of pellets was significantly lower than that of platelets and releasates, indicating the low level of α-granules and low platelet function in the pellet group (Fig. 1c). The mRNA levels of both EMT markers and ECM proteins indicated that pellets promoted MCF-7 EMT via direct interaction (Fig. 1d). Notably, to observe the secretion change of pellet after contacting MCF-7 cells, the MCF-7 cells were incubated again with the supernatant of the co-incubation of pellets and MCF-7 (the pellet+MCF-7 CM group). The result indicated that there was no significant difference between the releasate group and the pellet+MCF-7 CM group, suggesting that the secretion of pellets was not much changed after contacting MCF-7 cells.

### Platelets contact breast cancer cells via surface integrin α2β1

**Fig. 2:**
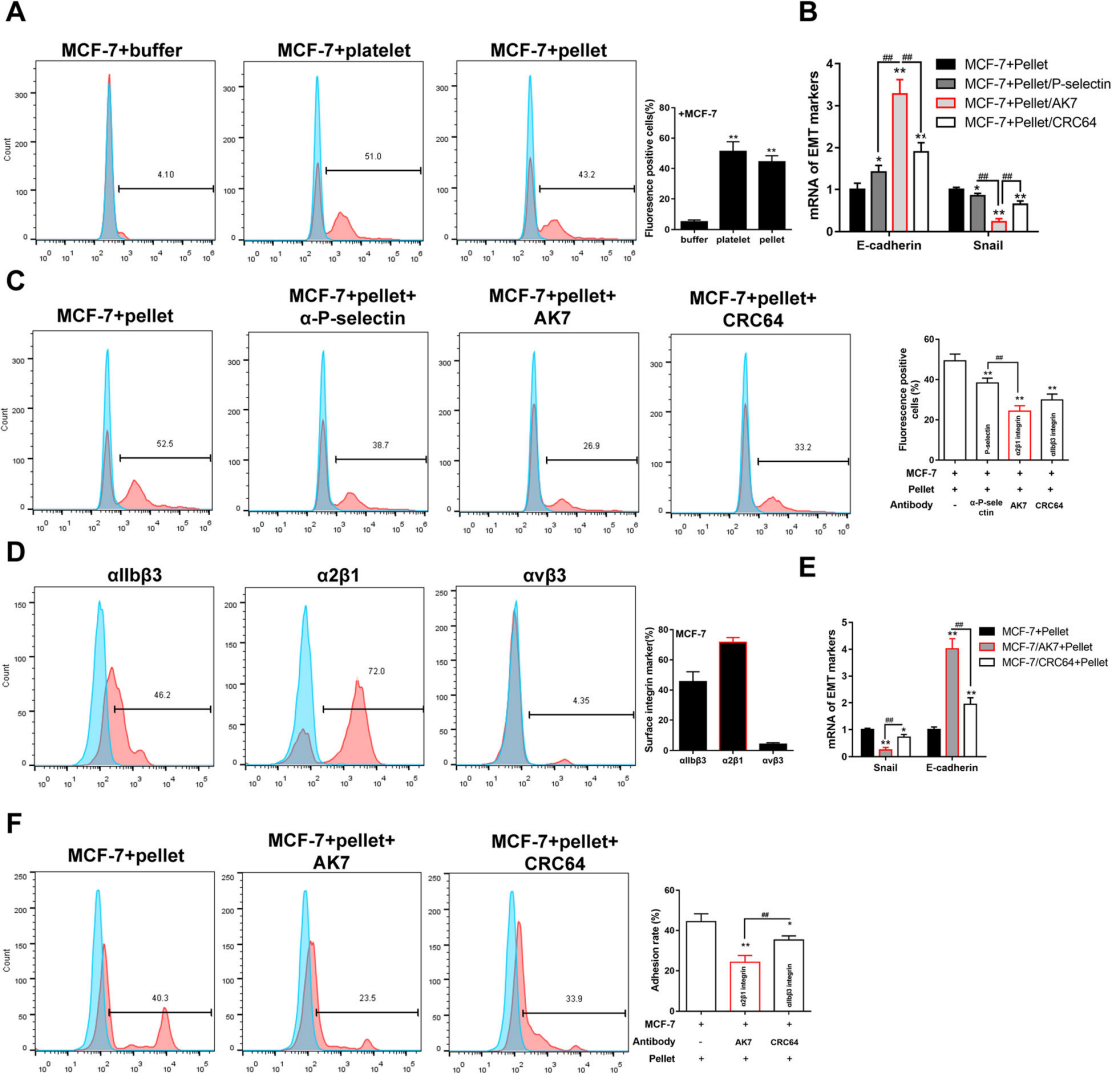
Surface integrin α2β1 mediated the contacting between platelet and MCF-7 cells. MCF-7 cells contacted with fluorescein-labeled platelets or pellets by the co-incubation for 30 min. **a** The percentage of fluorescein-positive MCF-7 cells was analyzed by the flow cytometry. Fluorescein-labeled pellets were co-incubated with MCF-7 cells after their surface proteins being inhibited by anti-P-selectin, anti-AK7, and anti-CRC64, the antibodies against P-selectin, integrin α2β1 and integrin αIIbβ3, respectively. **b** The relative mRNA levels of EMT markers in MCF-7 cells were determined by qRT-PCR and **c** the percentage of fluorescein-positive MCF-7 cells was analyzed by the flow cytometry. **d** The percentage of surface integrins on the MCF-7 cells. MCF-7 cells were co-incubated with fluorescein-labeled pellets after their surface proteins being inhibited by anti-AK7 and anti-CRC64. **e** The relative mRNA levels of EMT markers in MCF-7 cells were determined by qRT-PCR, and **f** the percentage of fluorescein-positive MCF-7 was analyzed by the flow cytometry. *p < 0.05, **p < 0.01, ^##^*p* < 0.01

MCF-7 cells were incubated with fluorescein-labeled platelets or pellets for 30 min, and the fluorescein-positive MCF-7 cells were analyzed by the flow cytometry to investigate the contacting ability between platelets and tumor cells. The result confirmed the enhancement of contacting ability between MCF-7 cells and platelets/pellets (Fig. 2a). Meanwhile, the antibodies, including anti-P-selectin, anti-AK7 (integrin α2β1), and anti-CRC64 (integrin αIIbβ3), were used to inhibit the surface proteins on pellets to explore the direct contacting site. The inhibition of the three pellet surface proteins markedly increased the mRNA level of E-cadherin and decreased the mRNA level of Snail. Interestingly, among the three proteins, the inhibition of integrin α2β1 most significantly changed the expression of EMT markers (Fig. 2b). Meanwhile, the pellet-MCF-7 contacting was dramatically suppressed by the inhibition of the three pellet surface proteins, especially by the inhibition of integrin α2β1 (Fig. 2c). On the MCF-7 cells, the integrin α2β1 and integrin αIIbβ3 had more proportions among the three surface integrins (Fig. 2d), therefore the anti-AK7 and anti-CRC64 were used to inhibit the surface proteins on MCF-7 cells. In the MCF-7 cells, the inhibition of the two surface integrins markedly increased the mRNA level of E-cadherin and decreased the mRNA level of Snail (Fig. 2e). In addition, the inhibition of integrin α2β1 most significantly changed the expression of EMT markers and inhibited MCF-7-pellet contacting (Fig. 2f). Another two breast cancer cell lines (MDA-MB-231 and SK-BR-3) were also used to co-incubate with platelets with or without inhibiting surface integrin α2β1. The results showed that the contacting ability was reduced after simultaneously inhibiting integrin α2β1 in platelets and tumor cells in comparison with uninhibited platelets + tumor cells (Additional file 1: Figure S1A). These data suggested that platelets contact breast cancer cells via surface integrin α2β1.

### Integrin α2β1 contacting between platelets and breast cancer cells activates Wnt-β-catenin pathway

**Fig. 3:**
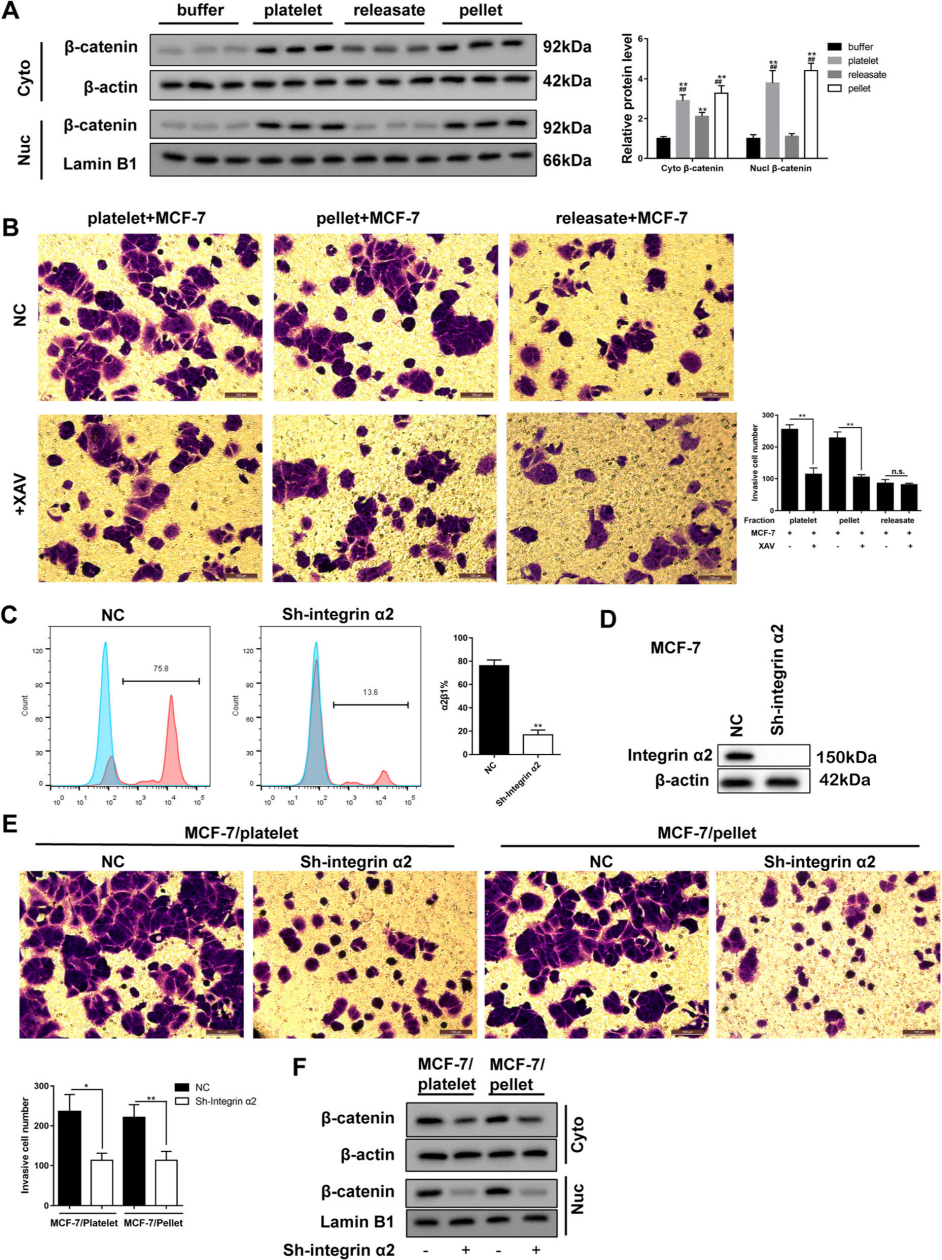
The effect of Wnt-β-catenin signaling and integrin α2β1-mediated contacting on the invasion of MCF-7 cells. **a** The expression of β-catenin protein in the cytoplasm and nucleus of the MCF-7 after co-incubation with platelets, releasates, and pellets was detected by western blotting. **b** The effect of platelets, releasates, and pellets on the MCF-7 cell invasion with or without the treatment of XAV, an inhibitor for Wnt-β-catenin. MCF-7 cells were transfected with Sh-integrin α2β1. Then the percentage of α2β1-positive MCF-7 cells (**c**), the expression of integrin α2β1 in MCF-7 cells (**d**), the number of invasive MCF-7 cells (**e**), and the expression of β-catenin in MCF-7 cytoplasm and nucleus (**f**) were detected. Scale bar = 100 μm. *p < 0.05, **p < 0.01, ^##^*p* < 0.01

We detected the expression of β-catenin in the MCF-7 cytoplasm and nucleus after pellet-MCF-7 contacting, and found that the expression of β-catenin in the MCF-7 nucleus was markedly increased after the platelet-MCF-7 and pellet-MCF-7 contacting (Fig. 3a). Meanwhile, the blockade of Wnt-β-catenin pathway by XAV dramatically decreased the number of invasive cells after the platelet-MCF-7 and pellet-MCF-7 contacting (Fig. 3b). Likewise, by integrin α2β1-silencing (Fig. 3c & d), the number of invasive MCF-7 cells, as well as the expression of β-catenin in MCF-7 cytoplasm and nucleus appeared a remarkable defect after the platelet-MCF-7 and pellet-MCF-7 contacting (Fig.  3e & f). In MDA-MB-231 cells and SK-BR-3 cells, the invasive cell numbers were significantly increased after the co-incubation of platelets and tumor cells, while such response was negated after the simultaneous inhibition of surface integrin α2β1 in platelets and tumor cells (Additional file 1: Figure S1B). Meanwhile, the simultaneous inhibition reduced the enhancement of β-catenin and Snail which were increased by the co-incubation of platelets and tumor cells (Additional file 1: Figure S1C). These data indicated that human breast cancer invasion was mediated by the platelet contacting in surface integrin α2β1, and subsequently activating Wnt-β-catenin pathway.

### Activated Wnt-β-catenin pathway promotes *tgfb1* transcription and TGF-β1 autocrine in MCF-7 cells

**Fig. 4:**
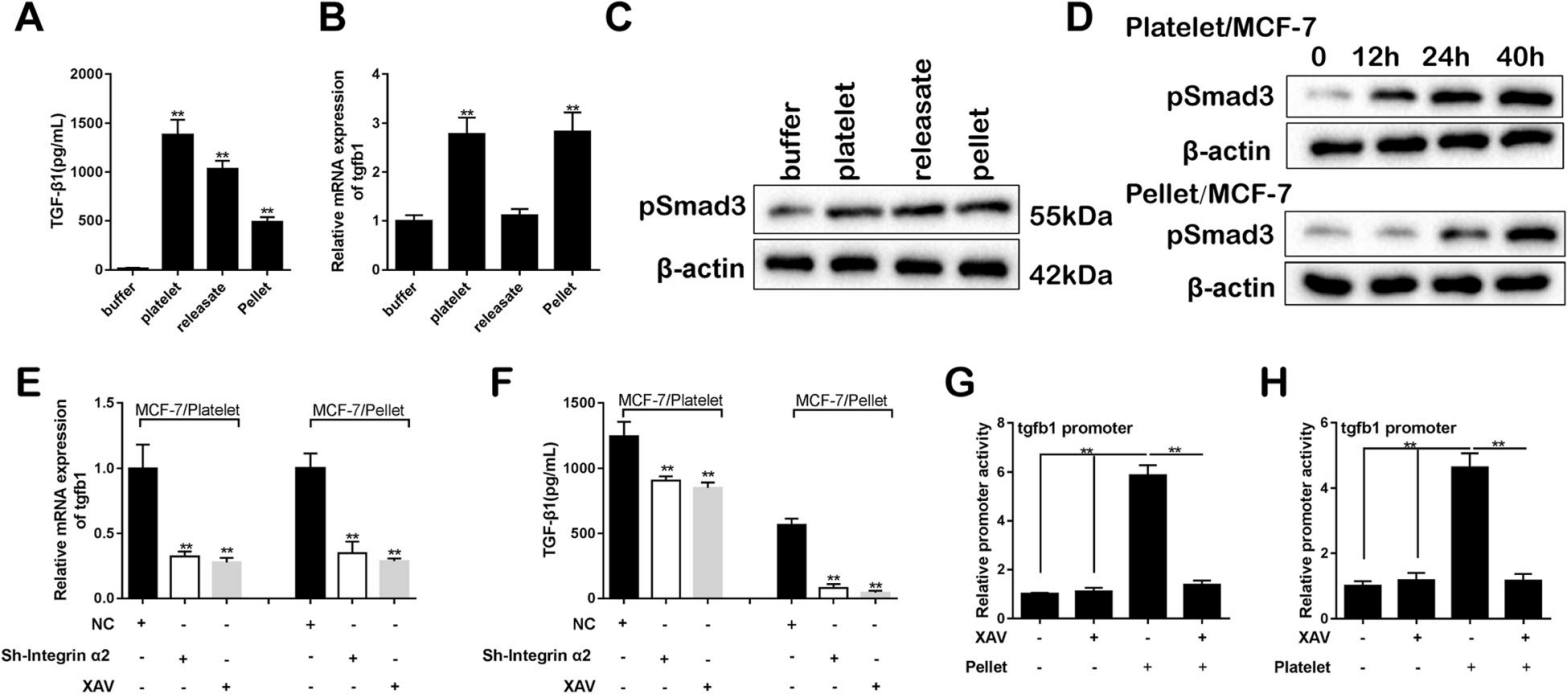
Activated Wnt-β-catenin signaling promotes *tgfb1* transcription and TGF-β1 autocrine in MCF-7 cells. The supernatant TGF-β1 level (**a**) and the *tgfb1* mRNA level (**b**) in MCF-7 cells after the co-incubation with platelets, releasates, or pellets. **c** The expression of pSmad3 protein, which is a downstream molecule of TGF-β1 activation, in MCF-7 cells. **d** The pSmad3 expression at 0, 12, 24, and 40 h after the platelet/MCF-7 co-incubation and the pellet/MCF-7 co-incubation. The *tgfb1* mRNA level (**e**), the supernatant TGF-β1 level (**f**), and the *tgfb1* promoter activity (**g** & **h**) were determined after integrin α2β1-silencing or the inhibition of Wnt-β-catenin. **p < 0.01

The supernatant TGF-β1 level, the *tgfb1* mRNA level and the *tgfb1* promoter activity after the pellet-MCF-7 contacting were detected to elucidate the effect of Wnt-β-catenin pathway activation. As shown in Fig. 4a, the supernatant TGF-β1 level was markedly increased by the contacting. The *tgfb1* mRNA level appeared an enhancement after the platelet-MCF-7 and pellet-MCF-7 contacting (Fig. 4b). Although the TGF-β1 level after the pellet-MCF-7 contacting seemed lower than that after the platelet-MCF-7 and the releasate-MCF-7 contacting, there was no significant difference in the expression of pSmad3, which is a downstream molecule of activated TGF-β1 (Fig. 4c). During the co-incubation between platelets and MCF-7 cells and the co-incubation between pellets and MCF-7 cells, the pSmad3 expression at 0, 12, 24, and 40 h was detected. With time increasing, the pSmad3 expression was gradually increased in both co-incubations, and the speed in the platelet/MCF-7 co-incubation seems faster than the pellet/MCF-7 co-incubation, whereas the pSmad3 expression at 40 h was not obviously different in the two groups (Fig. 4d). These data indicated that the pellet-induced TGF-β1 secretion could activate Smad3 signaling pathway. After integrin α2β1-silencing or Wnt-β-catenin blockade, both *tgfb1* mRNA level and TGF-β1 level were markedly reduced (Fig. 4e & f). Meanwhile, after the platelet-MCF-7 and pellet-MCF-7 contacting, the *tgfb1* promoter activity was significantly inhibited by Wnt-β-catenin blockade (Fig. 4g & h).

### Both Wnt-β-catenin and TGF-β1/pSmad3 pathways promote MCF-7 EMT

**Fig. 5:**
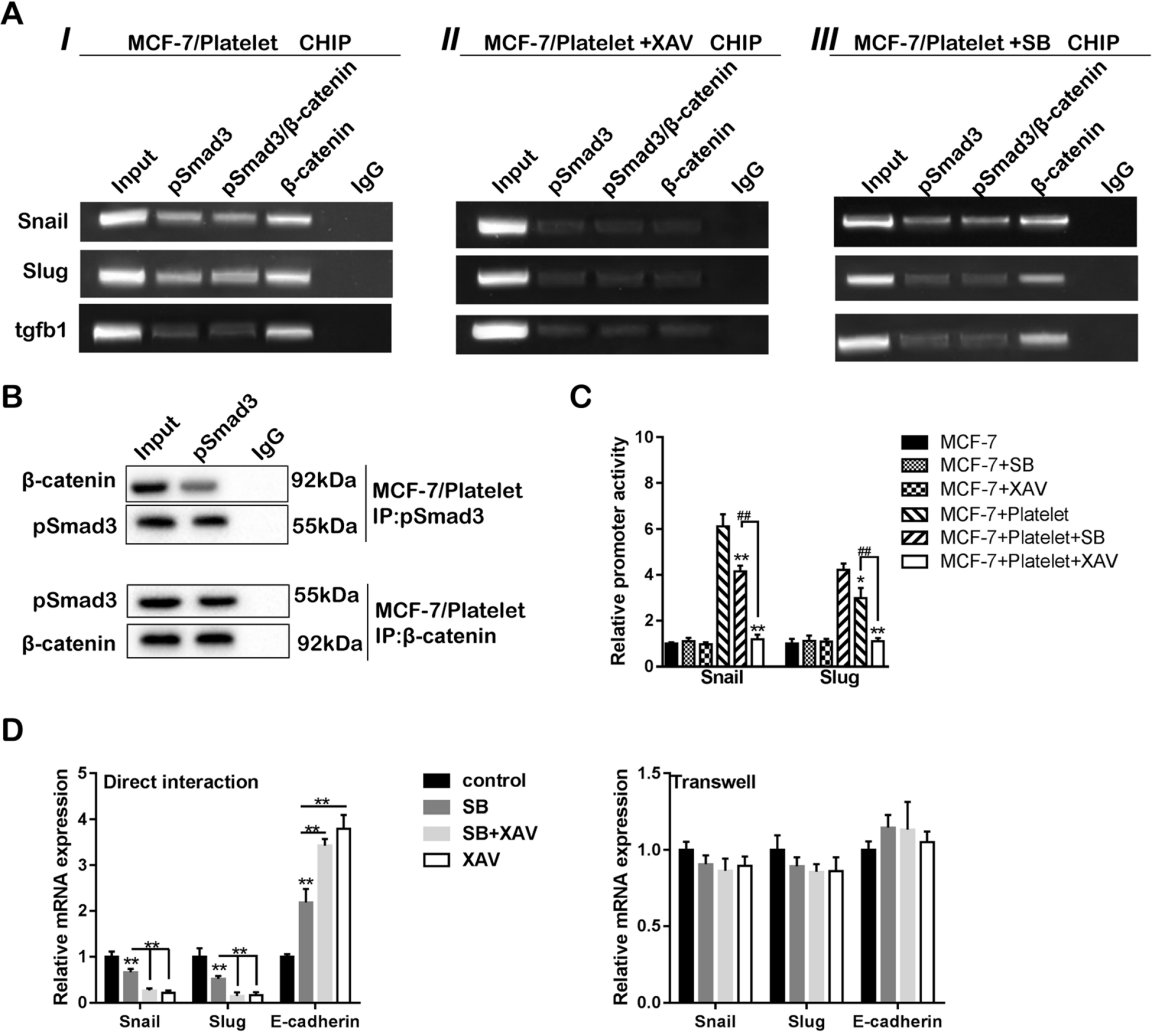
Both Wnt-β-catenin and TGF-β1/pSmad3 pathways promote MCF-7 cell EMT. **a** ChIP assay was performed to determine the combination between β-catenin/pSmad3 and the promoter of *Snail* and *Slug*. **b** The IP assay was performed to confirm the binding between β-catenin and pSmad3. **c** The promoter activity of *Snail* and *Slug* before and after the co-incubation with or without adding XAV, an inhibitor for β-catenin or SB, an inhibitor for pSmad3 pathway. **d** The mRNA expression of EMT markers was assessed in MCF-7 cells after the direct contacting and the transwell assay. *p < 0.05, **p < 0.01, ^##^*p* < 0.01

To explore the causal relationship between signaling pathways and EMT, ChIP and IP were performed in MCF-7 cells after the platelet-MCF-7 contacting. The ChIP result demonstrated that β-catenin and pSmad3 could interact with the promoter of *Snail* and *Slug* (Fig. 5aI). Blocking the Wnt-β-catenin pathway alone totally inhibited β-catenin and pSmad3 binding with the promoter of *Snail* and *Slug* (Fig. 5aII), while blocking the TGF-β1/pSmad3 pathway partly inhibited the interaction (Fig. 5aIII). As shown in Fig. 5b, IP confirmed the binding between β-catenin and pSmad3, indicating that TGF-β1/pSmad3 promoted *Snail* and *Slug* transcription via β-catenin and pSmad3 binding. The promoter activity of *Snail* and *Slug* was partly inhibited by TGF-β1/pSmad3 blockade, while it was greater inhibited by Wnt-β-catenin blockade (Fig. 5c). In comparison with the transwell invasion assay, the direct interaction between MCF-7 cells and platelets was more potent to MCF-7 EMT. Besides, Wnt-β-catenin pathway played a more important role than TGF-β1/pSmad3 pathway, as the EMT markers were more greatly changed after Wnt-β-catenin pathway blockade, but there seemed no difference between Wnt-β-catenin pathway blockade and blockade of both pathways (Fig. 5d).

### Integrin α2β1-silencing inhibits breast cancer metastasis in vivo

**Fig. 6:**
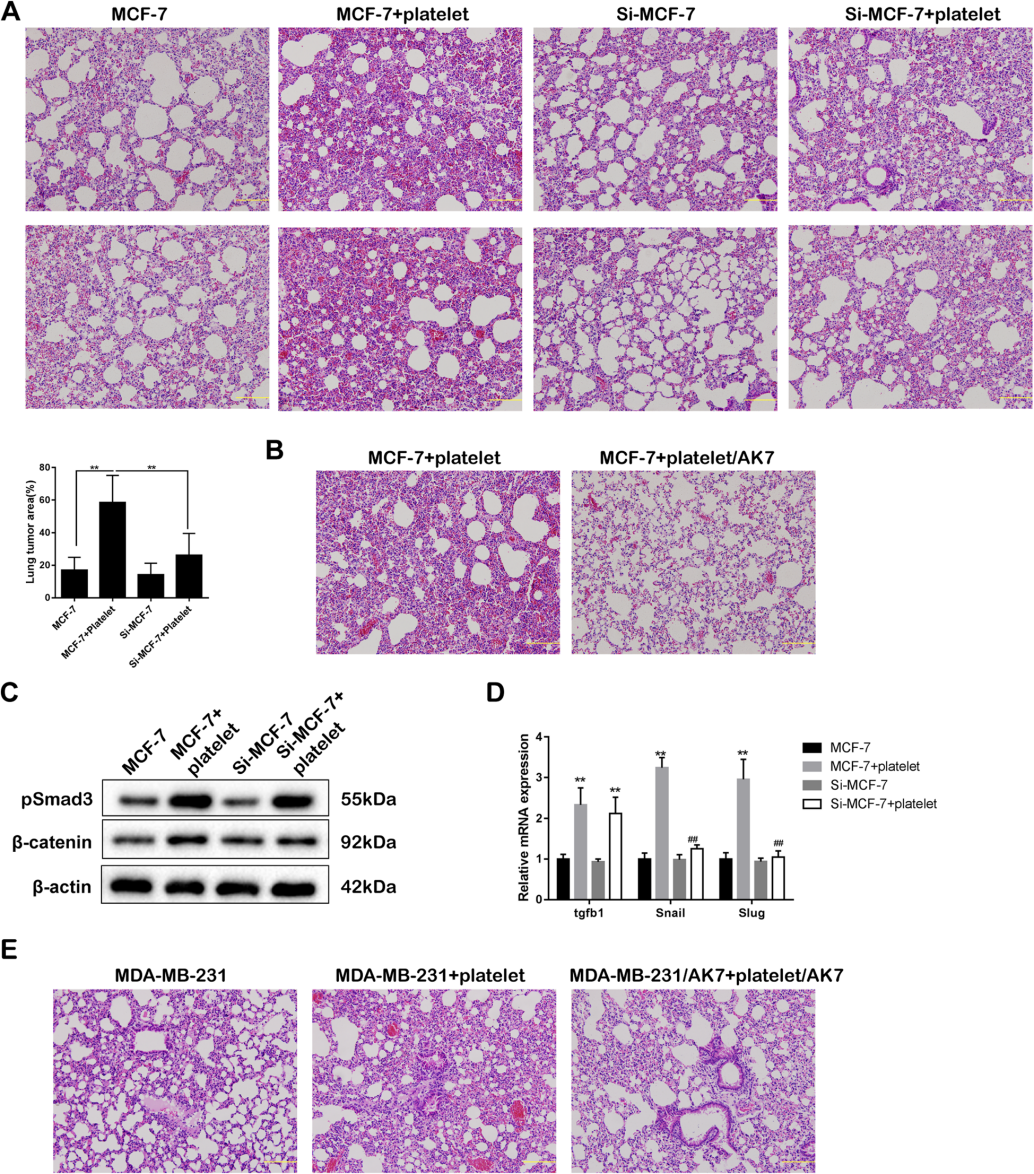
Integrin α2β1-silencing inhibits tumor cell metastasis in a mouse model for breast cancer lung metastasis*.* **a** The MCF-7 cells were transfected with the lentivirus vector containing interference sequences against integrin α2β1 to obtain the integrin α2β1-interfering MCF-7 cells (Si-MCF-7). Si-MCF-7 cells or MCF-7 cells (1.5 × 10^6^ cells/100 μl PBS) with or without co-incubating with platelets were intravenously injected into nude mice (*n* = 5 in each group). HE staining images and the percentage of lung tumor area were determined. **b** Following the treatment of anti-AK7 for 30 min, the platelets were co-incubated with MCF-7 cells for 40 h (*the MCF-7 + platelet/AK7 group*). The co-incubation between anti-AK7-untreated platelets and MCF-7 cells was the control (*the MCF-7 + platelet group*). The tumor cells (1.5 × 10^6^ cells/100 μl PBS) were intravenously injected into the lateral tail vein of immunodeficient nude mice (*n* = 5 in each group). **c** The protein expression of pSmad3 and β-catenin in lung tumors. **d** The mRNA expression of tgfb1, Snail and Slug in lung tumors. **e** MDA-MB-231 cells were co-incubated with platelets for 40 h (*the MDA-MB-231 + Platelet group*). Following the simultaneous inhibition of surface integrin α2β1, MDA-MB-231/AK7 cells were co-incubated with platelet/AK7 for 40 h (*the MDA-MB-231/AK7 + platelet/AK7 group*). MDA-MB-231 cells cultured in the normal medium was the control (*the MDA-MB-231 group*). The tumor cells (1 × 10^6^ cells/100 μl PBS) were intravenously injected into the lateral tail vein of immunodeficient nude mice (*n* = 5 in each group). Scale bar = 100 μm. **p < 0.01, ^##^*p* < 0.01

To investigate the effect of integrin α2β1-silencing in breast cancer metastasis, Si-MCF-7, conditioned MCF-7 cells with transfection of lentivirus vector containing interference sequences against integrin α2β1, were intravenously injected into nude mice to establish the mouse xenograft model. After grown for 5 weeks, the result of lung tissues staining by HE indicated the smaller tumor area in the Si-MCF-7 + platelet group than the MCF-7 + platelet group **(**Fig. 6a**)**. Less invasion was also observed in the MCF-7 + platelet/AK7 group in comparison with the MCF-7 + platelet group (Fig. 6b). In the lung tissues, both pSmad3 and β-catenin expressions in the MCF-7 + platelet group were increased in comparison with the MCF-7 group. There was a decrease in β-catenin expression in the Si-MCF-7 + platelet group compared with the MCF-7 + platelet group, although the difference of pSmad3 expression between the two groups was not significant (Fig. 6c). The mRNA expression of *tgfb1* was markedly increased in the MCF-7 + platelet group compared with the MCF-7 group, and in the Si-MCF-7 + platelet group compared with the Si-MCF-7 group. The mRNA expression of EMT markers was elevated in the MCF-7 + platelet group compared with the MCF-7 group, and in the Si-MCF-7 + platelet group compared with the MCF-7 + platelet group (Fig. 6d). On the other hand, the invasion area was increased in the MDA-MB-231 + platelet group, while it was reduced in the MDA-MB-231/AK7 + platelet/AK7 group (Fig. 6e). These data indicated that the direct contacting of surface integrin α2β1 between breast cancer cells and platelets increased tumor metastasis in vivo.

## Discussion

**Fig. 7:**
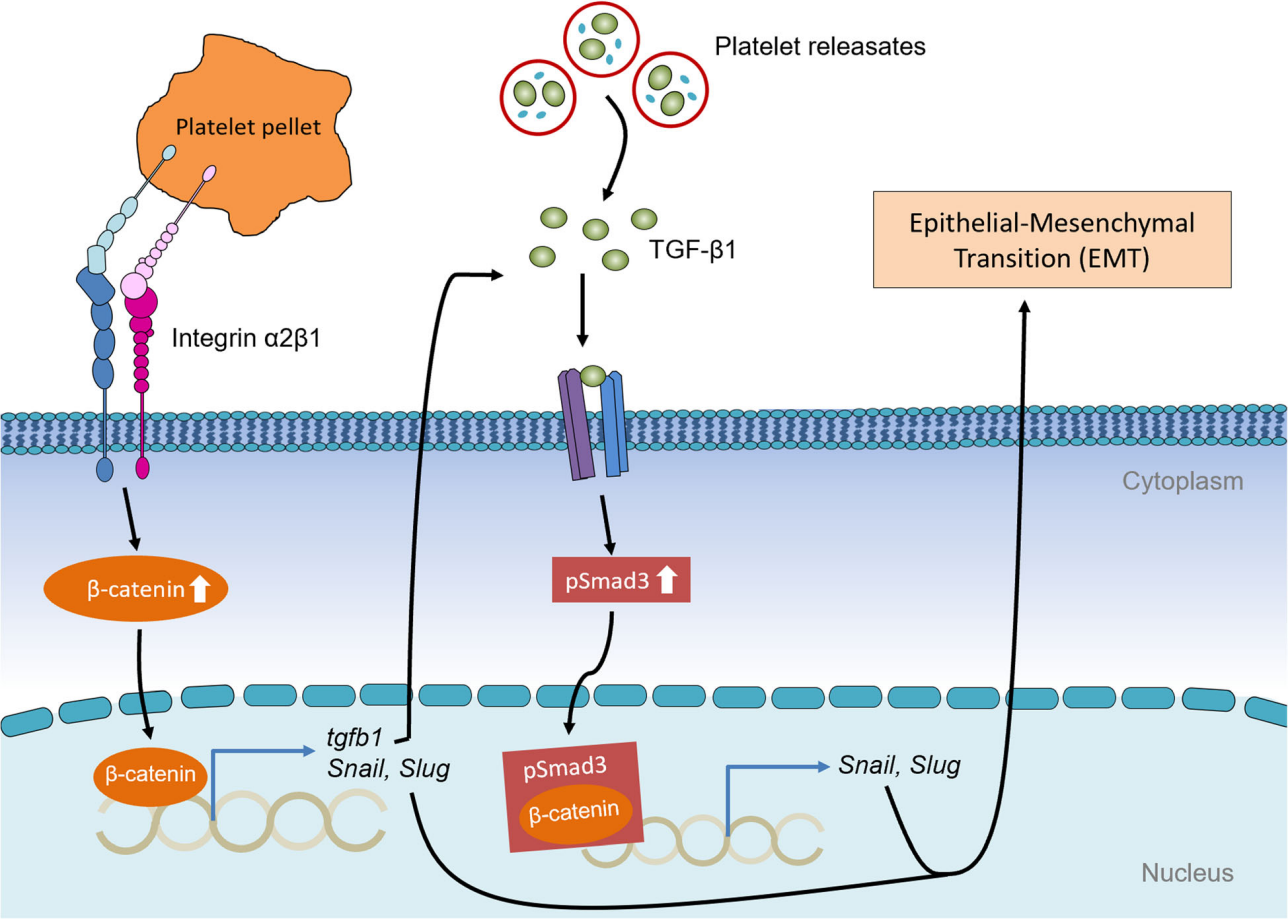
Platelets promote the EMT of breast cancer cell via surface integrin α2β1-mediated direct contacting. Surface integrin α2β1 mediated the direct contact between the MCF-7 cells and the platelet and promotes the activation of Wnt-β-catenin signaling pathway in MCF-7 cells. The activated Wnt-β-catenin signaling enhances the transcription of *tgfb1*, thereby promotes TGF-β1 secretion and the activation of the TGF-β1/pSmad3 pathway. Both β-catenin and the interaction of β-catenin and pSmad3 enhance the transcription of *Snail* and *Slug*, which are critical for the expression of EMT markers, thus promoting breast cancer metastasis

The current study demonstrated that the integrin α2β1 mediated the direct interaction between platelet and MCF-7 cells, thus promoting EMT and invasion of MCF-7 cells. In addition, by integrin α2β1 contacting, both the activation of Wnt-β-catenin pathway and the TGF-β1 autocrine increased the transcription of *Snail* and *Slug* (Fig. 7).

Accumulating studies have indicated the pro-metastatic function of platelets in various types of tumors, including cholangiocarcinoma [23], multiple myeloma [24], and ovarian cancer [9]. In breast cancer, the pro-metastatic function of platelets was initiated by the accumulation of platelets in primary tumor cells [7]. The mechanism of platelet accumulation is mainly related to the “First Responder” property of platelets in circulation [25]. Tumor cells with metastatic characteristics are often more aggressive, and easily enter the circulation, releasing direct signals that promote platelet aggregation [26]. Meanwhile, the aggregated platelets release angiogenic factors that exaggerate tumor growth and angiogenesis [27]. In the present work, we demonstrated the pro-metastatic function of platelets was initiated via coincubation of breast cancer cells with platelets and platelet fractions. Interestingly, our findings indicated that the direct interaction between tumor cells and pellets, or known as platelet membranes, plays the most important role in promoting MCF-7 cell invasion (Fig. 1), which is consistent with the former research [12]. Previous study indicated that releasates promote MDA-MB-231 cell (human breast cancer cell line) invasion [27], however, the pro-invasive effect of releasates to MCF-7 cells was low in our findings. We believed that it may be the potential mechanism of the lower metastatic property of MCF-7 cells than that of the MDA-MB-231 cells, although further investigations are still needed.

The importance of integrins that affect tumor progression has made them an appealing target for cancer therapy. Integrins are heterodimeric cell surface receptors that formed by the combination of 18 α-subunits and 8 β-subunits, mediating the contacting to the ECM and other molecules [16]. In breast cancer, integrin α6β4 is reported to increase the tumor size and grade, and decrease the survival rates [28, 29], while integrin αvβ3 is reported to promote bone metastasis of breast cancer patients [30, 31]. Meanwhile, tumor cell–platelet interactions mediated by integrins on the surfaces of both tumor cells and platelets are correlated with increased tumor metastasis. It is reported that the combined blockade of both tumor integrin αvβ3 and platelet integrin αIIbβ3 inhibited the angiogenesis and tumor growth of melanoma [17]. In the current study, we confirmed the high proportion of surface integrin α2β1 on MCF-7 cells, and revealed the reduced MCF-7 cell-pellets contacting ability after the blockade of tumor integrin α2β1 or pellet integrin α2β1 (Fig. 2), suggesting that antagonists that target integrin α2β1 on platelets or tumor cells may inhibit tumor invasion. Hence, we introduced integrin α2β1-silencing MCF-7 cells into the mouse xenograft model, and found the anti-metastatic effect of integrin α2β1-silencing. Although previous studies and our current work suggest that integrin α2β1 promotes metastasis of breast cancer, a study conducted by Ramirez et al. [32] indicated that integrin α2β1 acts as a metastasis suppressor in a mouse model of breast cancer. In their study, the number and size of metastatic foci in the lungs were significantly increased in the integrin α2-deficient mice in comparison with the wild type mice. We believed that apart from the platelet-related metastatic way, there may be other metastatic ways that mediated by integrin α2β1, which will be investigated in our further studies.

As reported, integrin α2β1 is a collagen receptor which can bind to type I, II, III, IV, and XI collagens [33]. Although little is known concerning the integrin α2β1-mediated cell contacting, a previous study revealed that the inhibition of the binding between integrin α2β1 and collagen suppressed platelet aggregation [34], suggesting collagen may mediate the binding between integrins. Plus, collagens are the essential component of ECM and provide both integrity and biological cues for cells [35]. We supposed that the direct binding between integrins in platelet and breast cancer cells was mediated by collagens, although more evidence is needed.

The Wnt-β-catenin signaling pathway is activated by cell surface receptors, and plays an important role in promoting EMT of breast cancer cells [36, 37]. Integrins are important cell surface receptors on tumor cells, and regulate the cellular β-catenin level, thus affecting transcriptions of target molecules. Cheng et al. revealed that the integrin β3/Wnt signaling mediate the chemoresistance and stemness of breast cancer cells [38]. Li et al. demonstrated that integrin α5/β-catenin signaling promotes the stemness and metastasis of triple negative breast cancer [39]. Based on these previous studies, we assessed the cytoplasm and nucleus levels of β-catenin in MCF-7 cells incubated with thrombin-activated platelets and pellets, and it appeared a significant enhancement of β-catenin in both cytoplasm and nucleus after contacting platelets and pellets (Fig. 3a). By integrin α2β1-silencing and Wnt-β-catenin blockade, we confirmed that the integrin α2β1/β-catenin pathway promotes MCF-7 cell invasion via increasing the expression of EMT correlated proteins Snail, Slug, and E-cadherin. Notably, the direct interaction between platelets and MCF-7 cells exerted more apparent influence on EMT correlated proteins than the transwell invasion assay (Fig. 5d), and we surmised that it may be related to the low invasive property of MCF-7 cells.

Except for the Wnt-β-catenin signaling pathway, the activation of the TGF-β1/Smad pathway in cancer cells also contributes to the EMT and metastasis of breast cancer [40]. Previous research demonstrated that the direct platelet-tumor cell contacting and platelet-derived TGF-β1 synergistically activate the TGF-β1/Smad pathway in cancer cells [13]. In our work, we found that both supernatant TGF-β1 and *tgfb1* mRNA levels were markedly enhanced after MCF-7 cell-platelet contacting, and the subsequently increased expression of pSmad3 was also confirmed. By integrin α2β1-silencing and Wnt-β-catenin blockade, we confirmed the activation of integrin α2β1/β-catenin/tgfb1 signaling cascade after MCF-7 cell-platelet/pellet contacting, indicating that the MCF-7 cells autocrine TGF-β1 after the contacting (Fig. 4). Moreover, we found that the TGF-β1/Smad pathway needs Wnt-β-catenin participation to regulate *Snail* and *Slug* transcriptions, as TGF-β1/pSmad3 blockade partly reduced transcription of *Snail* and *Slug*, while Wnt-β-catenin blockade almost cut off the transcription of *Snail* and *Slug* (Fig. 5). Combined with the IP results, we demonstrated that Wnt-β-catenin promotes EMT via dependent or independent of the TGF-β1/pSmad3 pathway.

## Conclusions

In conclusion, our findings indicated that the direct interaction between platelets and breast cancer cells exerts its pro-metastatic function via integrin α2β1 contacting and Wnt-β-catenin activation. The activated Wnt-β-catenin promotes the autocrine of TGF-β1 in MCF-7 cells. Meanwhile, both Wnt-β-catenin and TGF-β1/pSmad3 pathways promote the transcription of EMT related genes. Additionally, the integrin α2β1-silencing has the potential effect of inhibiting breast cancer metastasis, providing a novel target of treating breast cancer.

## Supplementary information

**Additional file 1:** **Figure S1.** The breast cancer cell lines (MDA-MB-231 and SK-BR-3) were used to co-incubate with platelets with or without inhibiting surface integrin α2β1. (A) The percentage of fluorescein-positive tumor cells was analyzed by the flow cytometry. (B) The number of invasive tumor cells. (C) The expression of β-catenin and Snail. Scale bar = 100 μm. **p < 0.01, ^##^*p* < 0.01.

## Data Availability

The datasets used and/or analyzed during the current study are available from the corresponding author on reasonable request.

## Abbreviations

ChIP: Chromatin Immunoprecipitation Assay
ECM: Extracellular matrix
EMT: Epithelial-mesenchymal transition
HE: Hematoxylin-eosin
PVDF: Poly-vinylidene difluoride
qRT-PCR: Quantitative Real-time PCR
SD: Standard deviation
TGF-β1: Transforming growth factor-β1

## References

[1] Mitch L (2010). Cell biology. Beyond clotting: the powers of platelets. Science.

[2] Bihari C (2016). Platelets contribute to growth and metastasis in hepatocellular carcinoma. Apmis.

[3] Levin J, Conley CL (1964). Thrombocytosis associated with malignant disease. Arch Intern Med.

[4] Watanabe A (2016). A novel clinical factor, D-dimer platelet multiplication, may predict postoperative recurrence and prognosis for patients with Cholangiocarcinoma. Ann Surg Oncol.

[5] Pardo L (2016). The prognostic value of pretreatment platelet count in patients with head and neck squamous cell carcinoma. Auris Nasus Larynx.

[6] Lam M (2017). The potential role of platelets in the consensus molecular subtypes of colorectal cancer. Cancer Metastasis Rev.

[7] Ishikawa S (2016). Platelets surrounding primary tumor cells are related to chemoresistance. Oncol Rep.

[8] Italiano JE (2008). Angiogenesis is regulated by a novel mechanism: pro- and antiangiogenic proteins are organized into separate platelet alpha granules and differentially released. Blood.

[9] Hu Q (2017). Role of platelet-derived Tgfβ1 in the progression of ovarian Cancer. Clin Cancer Res.

[10] Möhle R (1997). Constitutive production and thrombin-induced release of vascular endothelial growth factor by human megakaryocytes and platelets. Proc Natl Acad Sci U S A.

[11] He AD (2017). Platelet releasates promote the proliferation of hepatocellular carcinoma cells by suppressing the expression of KLF6. Sci Rep.

[12] Pang JH (2015). Activation of tumour cell ECM degradation by thrombin-activated platelet membranes: potentially a P-selectin and GPIIb/IIIa-dependent process. Clin Exp Metastasis.

[13] Labelle M, Begum S, Hynes RO (2011). Direct signaling between platelets and cancer cells induces an epithelial-mesenchymal-like transition and promotes metastasis. Cancer Cell.

[14] Hynes RO (2002). Integrins: bidirectional, allosteric signaling machines. Cell.

[15] Hynes RO (2002). A reevaluation of integrins as regulators of angiogenesis. Nat Med.

[16] Desgrosellier JS, Cheresh DA (2010). Integrins in cancer: biological implications and therapeutic opportunities. Nat Rev Cancer.

[17] Trikha M (2002). Multiple roles for platelet GPIIb/IIIa and αvβ3 Integrins in tumor growth, angiogenesis, and metastasis. Cancer Res.

[18] Shou L-M (2013). Cantharidin and norcantharidin inhibit the ability of MCF-7 cells to adhere to platelets via protein kinase C pathway-dependent downregulation of α2 integrin. Oncol Rep.

[19] Morozevich GE (2015). Implication of α2β1 integrin in anoikis of MCF-7 human breast carcinoma cells. Biochem Mosc.

[20] Yeh HW (2018). PSPC1 mediates TGF-β1 autocrine signalling and Smad2/3 target switching to promote EMT, stemness and metastasis. Nat Cell Biol.

[21] Qijin L, Malinauskas RA (2015). Comparison of two platelet activation markers using flow cytometry after in vitro shear stress exposure of whole human blood. Artif Organs.

[22] Gomes FG (2017). Breast-cancer extracellular vesicles induce platelet activation and aggregation by tissue factor-independent and -dependent mechanisms. Thromb Res.

[23] Cadamuro M (2019). Platelet-derived growth factor-D enables liver myofibroblasts to promote tumor lymphangiogenesis in cholangiocarcinoma. J Hepatol.

[24] Takagi S (2018). Platelets enhance multiple myeloma progression via IL-1β Upregulation. Clin Cancer Res.

[25] Menter DG (2017). Platelet "first responders" in wound response, cancer, and metastasis. Cancer Metastasis Rev.

[26] Labelle M, Hynes RO (2012). The initial hours of metastasis: the importance of cooperative host-tumor cell interactions during hematogenous dissemination. Cancer Discov.

[27] Jiang L (2017). Platelet releasate promotes breast cancer growth and angiogenesis via VEGF-integrin cooperative signalling. Br J Cancer.

[28] Diaz LK (2005). β4 integrin subunit gene expression correlates with tumor size and nuclear grade in early breast cancer. Mod Pathol.

[29] Friedrichs K (1995). High expression level of α6 integrin in human breast carcinoma is correlated with reduced survival. Cancer Res.

[30] Takayama S (2005). The relationship between bone metastasis from human breast cancer and integrin alpha(v)beta3 expression. Anticancer Res.

[31] Liapis H, Flath A, Kitazawa S (1996). Integrin alpha V beta 3 expression by bone-residing breast cancer metastases. Diagn Mol Pathol.

[32] Ramirez NE (2011). The alpha (2) beta (1) integrin is a metastasis suppressor in mouse models and human cancer. J Clin Invest.

[33] Erpenbeck L, Schon MP (2010). Deadly allies: the fatal interplay between platelets and metastasizing cancer cells. Blood.

[34] Momic T (2015). Vipegitide: a folded peptidomimetic partial antagonist of alpha2beta1 integrin with antiplatelet aggregation activity. Drug Des Devel Ther.

[35] Bardsley K, Yang Y, El Haj AJ (2017). Fluorescent labeling of collagen production by cells for noninvasive imaging of extracellular matrix deposition. Tissue Eng Part C Methods.

[36] Xie SL (2017). SOX8 regulates cancer stem-like properties and cisplatin-induced EMT in tongue squamous cell carcinoma by acting on the Wnt/β-catenin pathway. Int J Cancer.

[37] Nastaran Mohammadi G, Sadegh B (2015). Interplay between microRNAs and WNT/β-catenin signalling pathway regulates epithelial-mesenchymal transition in cancer. Eur J Cancer.

[38] Cheng S (2019). FSTL1 enhances chemoresistance and maintains stemness in breast cancer cells via integrin β3/Wnt signaling under miR-137 regulation. Cancer Biol Ther.

[39] Li Y (2019). In vivo β-catenin attenuation by the integrin α5-targeting nano-delivery strategy suppresses triple negative breast cancer stemness and metastasis. Biomaterials.

[40] Zhao S-J (2018). CD151 promotes breast cancer metastasis by activating TGF-β1/Smad signaling pathway. Eur Rev Med Pharmacol Sci.

