# Correction to: Platelets promote breast cancer cell MCF-7 metastasis by direct interaction: surface integrin α2β1-contacting-mediated activation of Wnt-β-catenin pathway

**DOI:** 10.1186/s12964-020-00641-7

**Published:** 2020-08-13

**Authors:** Xiao-xiao Zuo, Ya Yang, Yue Zhang, Zhi-gang Zhang, Xiao-fei Wang, Yong-gang Shi

**Affiliations:** Department of Radiation Oncology, The First Affiliated Hospital of Zhengzhou University, No.1 East Jianshe Road, Erqi District, Zhengzhou, Henan Province 450000 People’s Republic of China

**Correction to: Cell Commun Signal 17, 142 (2019)**

**https://doi.org/10.1186/s12964-019-0464-x**

Following publication of the original article [1], the authors identified an error in the Fig. 3b. The images in the “releasate + MCF-7” group were incorrect. The updated and corrected Fig. 3 is given in this correction article.

**Fig. 3:**
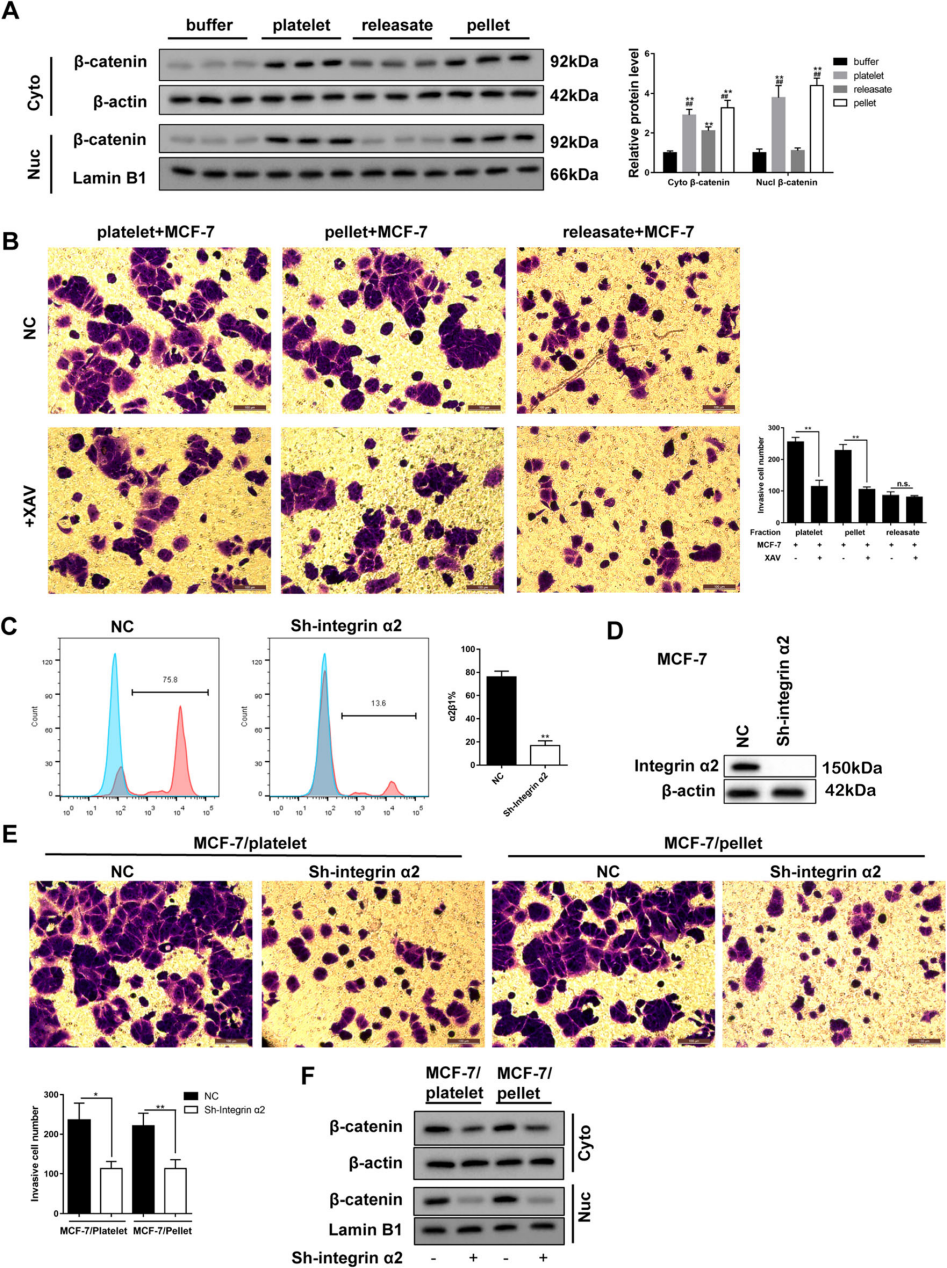
The effect of Wnt-β-catenin signaling and integrin α2β1-mediated contacting on the invasion of MCF-7 cells. **a** The expression of β-catenin protein in the cytoplasm and nucleus of the MCF-7 after co-incubation with platelets, releasates, and pellets was detected by western blotting. **b** The effect of platelets, releasates, and pellets on the MCF-7 cell invasion with or without the treatment of XAV, an inhibitor for Wnt-β-catenin. MCF-7 cells were transfected with Sh-integrin α2β1. Then the percentage of α2β1-positive MCF-7 cells (**c**), the expression of integrin α2β1 in MCF-7 cells (**d**), the number of invasive MCF-7 cells (**e**), and the expression of β-catenin in MCF-7 cytoplasm and nucleus (**f**) were detected. Scale bar = 100 μm. *p < 0.05, **p < 0.01, ^##^*p* < 0.01
