# Supplementary figures and images for: Platelets promote breast cancer cell MCF-7 metastasis by direct interaction: surface integrin α2β1-contacting-mediated activation of Wnt-β-catenin pathway
Source: Cell Commun Signal. 2019 Nov 7;17:142. doi: 10.1186/s12964-019-0464-x (PMC6836423; doi:10.1186/s12964-019-0464-x)

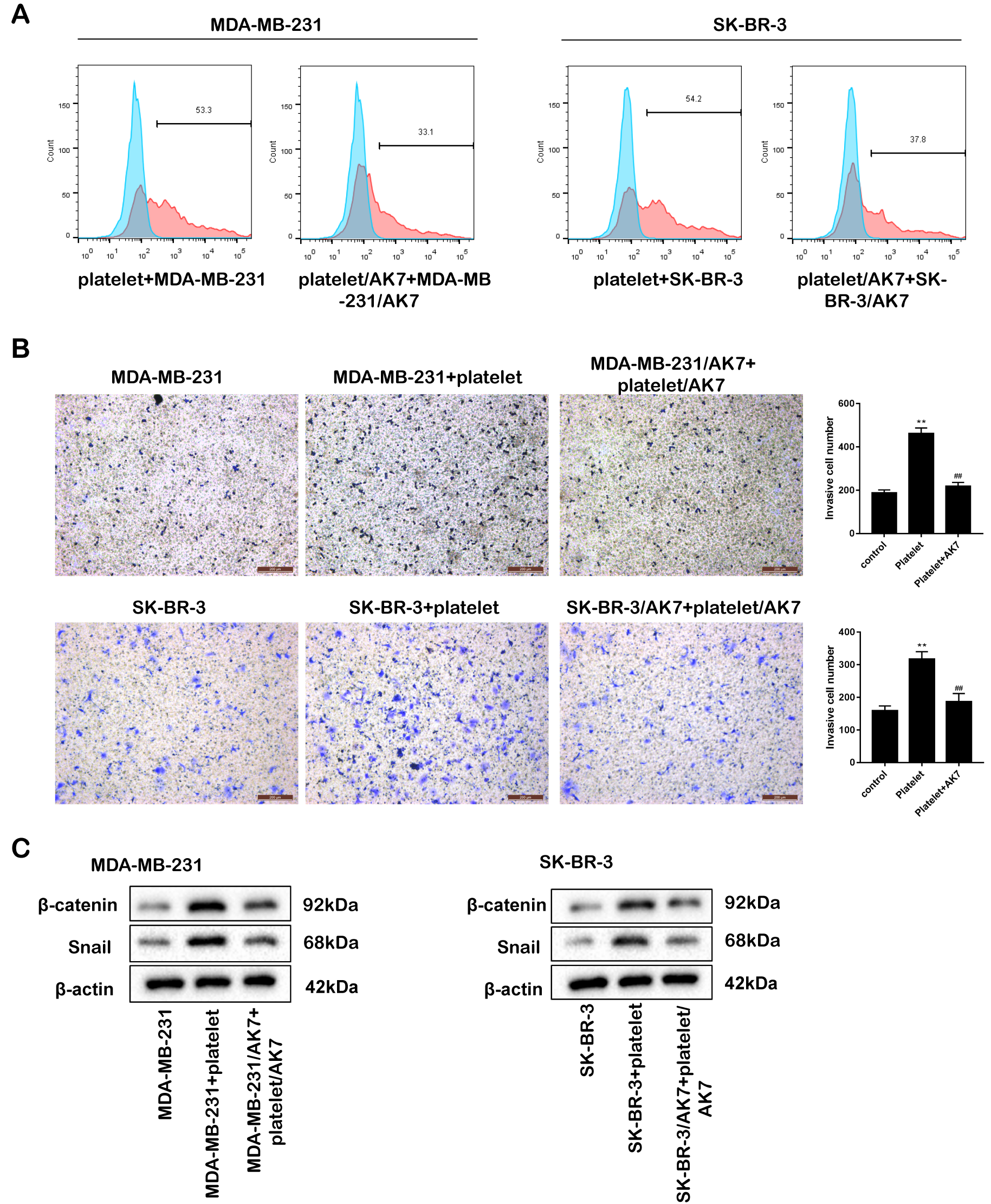

Supplement: Supplementary file 1 — Additional file 1: Figure S1. The breast cancer cell lines (MDA-MB-231 and SK-BR-3) were used to co-incubate with platelets with or without inhibiting surface integrin α2β1. (A) The percentage of fluorescein-positive tumor cells was analyzed by the flow cytometry. (B) The number of invasive tumor cells. (C) The expression of β-catenin and Snail. Scale bar = 100 μm. **p < 0.01, ##p < 0.01. [file 12964_2019_464_MOESM1_ESM.tif]
